# Mortality in Patients with Acute Coronary Syndrome—A Prospective 5-Year Follow-Up Study

**DOI:** 10.3390/jcm12206598

**Published:** 2023-10-18

**Authors:** Thuy Mi Nguyen, Daniela Melichova, Eivind W. Aabel, Øyvind H. Lie, Lars Gunnar Klæboe, Bjørnar Grenne, Benthe Sjøli, Harald Brunvand, Kristina Haugaa, Thor Edvardsen

**Affiliations:** 1 Department of Cardiology, Hospital of Southern of Norway, 4604 Kristiansand, Norway; thmngu@ous-hf.no (T.M.N.); daniela.melichova@sshf.no (D.M.); benthe.sjoli@sshf.no (B.S.); harbrun@online.no (H.B.); 2 ProCardio, Center for Innovation, Department of Cardiology, Oslo University Hospital, Rikshospitalet, 0424 Oslo, Norway; b32129@ous-hf.no (E.W.A.); b28176@ous-hf.no (Ø.H.L.); lgklaeboe@yahoo.no (L.G.K.); kristina.haugaa@medisin.uio.no (K.H.); 3 Institute of Clinical Medicine, Faculty of Medicine, University of Oslo, 0318 Oslo, Norway; 4 Centre for Innovative Ultrasound Solutions and Department of Circulation and Medical Imaging, Norwegian University of Science and Technology, 7491 Trondheim, Norway; bjornar.grenne@ntnu.no; 5 Clinic of Cardiology, St Olavs Hospital, 7006 Trondheim, Norway

**Keywords:** acute myocardial infarction, long-term follow up, mortality, non-ST-segment elevation myocardial infarction, ST-segment elevation myocardial infarction

## Abstract

Our objective was to compare long-term outcomes in patients with non-ST-elevation myocardial infarction (NSTEMI) and ST-elevation myocardial infarction (STEMI) between two time periods in Southern Norway. There are limited contemporary data comparing long-term follow-up after revascularization in the last decades. This prospective follow-up study consecutively included both NSTEMI and STEMI patients during two time periods, 2014–2015 and 2004–2009. Patients were followed up for a period of 5 years. The primary outcome was all-cause mortality after 1 and 5 years. A total of 539 patients with acute myocardial infarction (AMI), 316 with NSTEMI (234 included in 2014 and 82 included in 2007) and 223 with STEMI (160 included in 2014 and 63 included in 2004). Mortality after NSTEMI was high and remained unchanged during the two time periods (mortality rate at 1 year: 3.5% versus 4.9%, *p* = 0.50; and 5 years: 11.4% versus 14.6%, *p* = 0.40). Among STEMI patients, all-cause mortality at 1 year was reduced in 2014 compared to 2004 (1.3% versus 11.1%, *p* < 0.001; and 5 years: 7.0% versus 22.2%, *p* = 0.004, respectively). Time to coronary angiography in NSTEMI patients remained unchanged between 2014 and 2007 (28.2 h [IQR 18.1–46.3] versus 30.3 h [IQR 18.0–48.3], *p* = 0.20), while time to coronary angiography in STEMI patients was improved in 2014 compared with 2004 (2.8 h [IQR 2.0–4.8] versus 21.7 h [IQR 5.4–27.1], *p* < 0.001), respectively. During one decade of AMI treatment, mortality in patients with NSTEMI remained unchanged while mortality in STEMI patients decreased, both at 1 and 5 years.

## 1. Introduction

Outcomes among patients presenting with ST–elevation myocardial infarction (STEMI) have improved markedly during the last two decades because of a combination of improved prehospital triage, early invasive revascularization with percutaneous coronary intervention (PCI) [1] and aggressive secondary preventive treatment strategies [2]. However, some studies suggest that short- and long-term outcomes have not improved in patients with non-ST-segment elevation myocardial infarction (NSTEMI) at the same rate seen in STEMI patients, possibly reflecting NSTEMI patients’ more complex clinical phenotype, including older age, more comorbidity and higher likelihood of a previous MI and recurrent ischemia [2]. A subset of patients with total occlusion present as NSTEMI without classic ST-elevation on the electrocardiogram (ECG). This may lead to delay in identification of these patients and further management. However, it has also been reported that the time interval between the onset of symptoms and treatment is longer in patients with NSTEMI [3], and that they are less likely to receive guideline-recommended treatment strategies at discharge [4,5].

Changes in survival for NSTEMI and STEMI during the last decades have so far been studied in registries or trials with short follow-up times [3,6,7,8]. Nevertheless, limited recent data are available describing long-term outcomes in prospective data series. Therefore, in this prospective study, we aimed to compare and describe long-term mortality in patients within NSTEMI and STEMI at two different time periods in Southern Norway, with long transport distances between community hospitals and tertiary PCI centers.

## 2. Materials and Methods

### 2.1. Study Design, Recruitment and Patient Population

The study population with acute myocardial infarction (AMI) was enrolled during two time periods in a local hospital in Southern Norway, 2014–2015 and 2004–2009. The study patients from 2014 were part of a prospective, observational, multicenter sub-study of the Norwegian IMPROVE study [9] (NCT02286908). NSTEMI from 2007 was part of the Echo-STRACS [10] study, where patients admitted with suspected acute coronary syndrome were consecutively evaluated for study inclusion. Patients from 2004 consisted of selected patients with first-time acute STEMI treated with thrombolysis and rescue PCI [11].

The inclusion criteria were age ≥ 18 years with NSTEMI or STEMI, based on typical symptoms, electrocardiogram, and cardiac biomarkers [12,13]. We excluded patients with significant valvular dysfunction or patients undergoing ventricular pacing. All patients were treated systematically according to the current guidelines at the time of inclusion [13]. Patient characteristics and biochemical work-up were prospectively obtained from hospital records. Killip classification at admittance and Global Registry of Acute Coronary Event (GRACE) risk score were calculated for all patients.

The primary outcome was death from any cause within 5 years. The secondary outcomes were reinfarction, defined as chest pain with rises or falls of cardiac biomarkers with or without changes on the ECG which required invasive intervention, and heart failure defined as new onset or worsening of clinical heart failure, with pulmonary congestion and in need of any treatment. Outcome data were collected from patients’ electronic health records at the hospital, which were linked with the governmental death registry database. Furthermore, we examined both unadjusted and multivariable adjusted cardiovascular risk factors to investigate differential predictors of mortality.

### 2.2. Coronary Angiography

All patients underwent coronary angiography on clinical indication in the acute phase of MI in the two tertiary PCI centers. Coronary angiography was performed by standard technique, using digital image acquisition and in accordance with clinical practice guidelines [14]. The PCI was carried out by experienced operators at the invasive centers. Importantly, the choice of stent types for angioplasty was not predetermined by the study protocol; instead, it was determined by the availability and suitability of stents as assessed by the cardiologists at the PCI center. The selection was made based on the severity of coronary blockage and clinical judgement of the interventional cardiologist. Procedural success rate was not a predetermined part of the study protocol but was reported in conjunction with a procedure success endpoint. STEMIs from 2004 patients were treated first with thrombolysis and received rescue PCI in case of recurrent unstable angina after thrombolysis. Coronary angiography for the remaining patients in this group was performed when judged appropriate given patients’ clinical pictures during the same hospitalization. If coronary artery bypass graft (CABG) was recommended as means of revascularization, surgery was performed as soon as possible.

### 2.3. Echocardiography

Echocardiographic examinations were performed after invasive procedures and before discharge, approximately 2–3 days after MI. The study examinations were performed using the Vivid 7 and Vivid 9 system (GE Vingmed Ultrasound AS, Horten, Norway) and analyzed using commercially available software (EchoPAC v202, GE Healthcare, Horten, Norway). Three consecutive cycles in 3 apical planes and 3 short-axis planes were obtained by conventional 2D gray-scale echocardiography, using second harmonic imaging. Loops were digitally stored and later analyzed off-line. Left ventricular ejection fraction (LVEF) was calculated from 4-chamber and 2-chamber images, using the modified Simpson rule. Valvular dysfunction was defined according to guidelines [15].

### 2.4. Statistical Analyses

Categorical data were presented as numbers and percentages and continuous data as mean ± SD or median (interquartile range). Comparisons of means were made using Student’s *t*-test and Mann–Whitney U-test, as appropriate. Kaplan–Meier survival analysis was used to estimate proportion of patients free from all-cause mortality rates, and groups were compared using log-rank test. Univariable Cox proportional hazard regression was used to identify potential markers of all-cause death at 5 years, and multivariable analysis included significant (*p* < 0.05) variables from the univariable analyses. Separate models were created for AMI risk factors and for clinical features at admission. Furthermore, separate models were created for recurrent MI and heart failure hospitalization and time to coronary angiography. We used log base-10 transformation of troponin T, NT-pro BNP and time to revascularization to meet model linearity assumptions. A *p*-value < 0.05 was considered significant. Statistical analyses were performed using IBM SPSS Statistics 26.0 (IBM Corp., Armonk, NY, USA).

## 3. Results

### 3.1. Study Population

**Figure 1:**
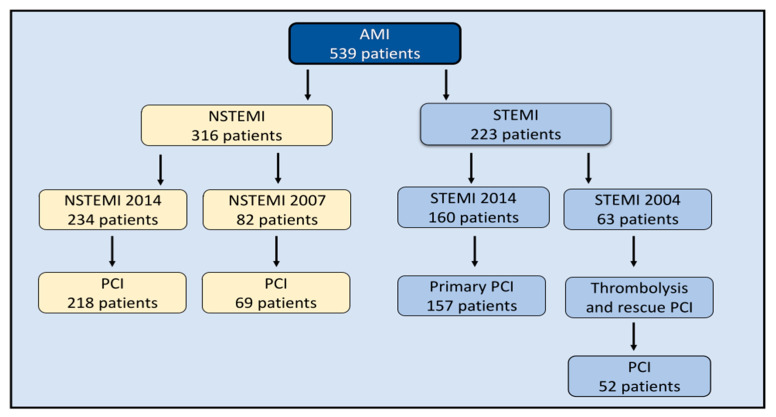
Study population. Flow chart demonstrating inclusion of patients during index hospitalization. Acute myocardial infarction (AMI), non-ST elevation myocardial infarction (NSTEMI), percutaneous coronary intervention (PCI) and ST-elevation myocardial infarction (STEMI).

We included a total of 539 patients with AMI, 316 patients with NSTEMI and 223 patients with STEMI (Figure 1). Among the NSTEMI patients, 234 patients were included in 2014–2015 (NSTEMI 2014) and 82 patients were included in 2007–2009 (NSTEMI 2007). Among the STEMI patients, 160 patients were included in 2014–2015 (STEMI 2014) and 63 patients were included in 2004 (STEMI 2004). All patients underwent coronary angiography, 97% of NSTEMI patients received PCI and 98% of STEMI 2014 patients in were treated with primary PCI. STEMI 2004 patients were treated with initial thrombolysis due to long transport distances and all patients went to coronary angiography for rescue PCI or CABG (*n* = 5) before discharge. None was lost to follow-up. The study populations were similar in terms of age, sex and comorbidities (Table 1).

### 3.2. Non-ST Elevation Myocardial Infarction: Comparisons between 2014 and 2007 Cohorts

The NSTEMI 2014 patients were more likely to have risk factors like higher age and history of coronary artery disease compared to NSTEMI 2007 patients (Table 1). The NSTEMI 2014 patients more frequently used angiotensin-converting enzyme inhibitors or angiotensin receptor blockers, whereas they less frequently used b-blockers and statins. Neither troponin T nor LVEF before discharge differed between NSTEMI 2014 and NSTEMI 2007 patients.

The rate of revascularization was similar in the two NSTEMI groups, and there were no differences in patients without intervention (Table 2). However, the method of revascularization was different, as the PCI rate was higher in NSTEMI 2014 patients, whereas CABG was more common in NSTEMI 2007. The median time from symptom onset to coronary angiography remained unchanged between NSTEMI 2014 and NSTEMI 2007. The frequency of occluded infarct-related artery in the two groups was similar, and there were no significant differences in localization of culprit lesions.

### 3.3. ST Elevation Myocardial Infarction Comparisons between 2014 and 2004 Cohorts

Overall, baseline characteristics were similar in the STEMI 2014 and STEMI 2004 patients. The baseline angiographic and procedural characteristics of STEMI patients are listed in Table 3. Not surprisingly, the STEMI 2014 cohort had shorter time to PCI compared with STEMI 2004. Only 17 of 63 of patients (27%) in STEMI 2004 went directly to rescue PCI after thrombolysis. After thrombolysis, 25 patients (40%) had remaining occlusion of the infarct-related artery at the time of angiography. A culprit lesion was identified in 60 of 63 patients (95%) after thrombolysis in STEMI 2004. Of these, 52 patients (84%) received PCI under the same procedure and 5 patients (8%) underwent CABG. Patients were more likely to undergo PCI in STEMI 2014 than STEMI 2004. The primary reasons for not performing PCI in both groups were the absence of a culprit lesion or the identification of a lesion that was not amenable.

### 3.4. Primary Outcome: All-Cause Mortality

**Figure 2:**
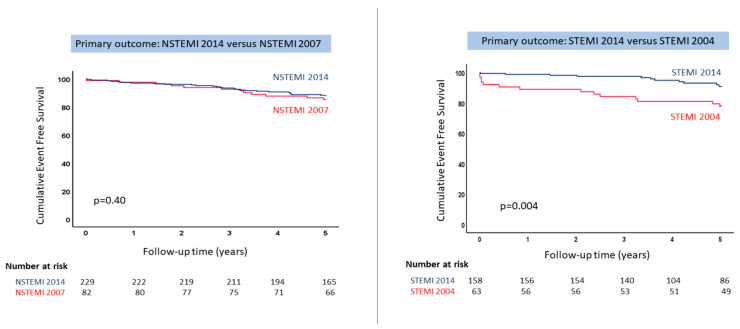
Cumulative survival in NSTEMI and STEMI patients by time period. Kaplan–Meier survival according to STEMI and NSTEMI for 5 years follow-up period. NSTEMI = non-ST-elevation myocardial infarction; STEMI = ST-elevation myocardial infarction.

In patients with NSTEMI, mortality remained unchanged in NSTEMI 2014 and in NSTEMI 2007 both for 1-year all-cause mortality and 5-year all-cause mortality, respectively (Figure 2). There was similar 5-year all-cause mortality in NSTEMI patients with occluded coronary arteries compared to those with stenosis.

In patients with STEMI, mortality was lower in STEMI 2014 patients treated with primary PCI compared to STEMI 2004 treated with thrombolysis and rescue PCI (Figure 2), with a 1- and 5-year mortality rate that decreased (respectively).

### 3.5. Secondary Outcome: Heart Failure Hospitalization and Reinfarction

During the 5-year follow-up period, rate of reinfarction was similar in the two NSTEMI populations. However, the rate of heart failure hospitalization decreased in NSTEMI 2014 patients compared with NSTEMI 2004 patients (Table 2).

The occurrence of recurrent AMI and rehospitalization for heart failure was significantly decreased in the STEMI 2014 patients compared with STEMI 2004 patients, respectively (Table 3).

### 3.6. Predictors of 5-Year Mortality in NSTEMI and STEMI

Variables predicting mortality are presented in Table 4. In multivariable analyses, independent predictors of increased 5-year mortality in NSTEMI were higher age, history of CAD, higher GRACE risk score, lower LVEF, time from hospitalization to PCI and time from symptoms to PCI.

In the STEMI population, independent predictors in multivariable analyses were older age, higher GRACE risk score, higher NT-proBNP, higher troponin T, time from hospitalization to PCI and time from symptoms to PCI.

## 4. Discussion

The main finding of our study was that after one decade, patients with NSTEMI all-cause mortality in 1 and 5 years remained unchanged while all-cause mortality in STEMI patients decreased. The rate of reinfarction remained unchanged in NSTEMI, while the rate of heart failure rehospitalizations decreased. In STEMI patients, both occurrence of reinfarction and heart failure rehospitalizations decreased from 2004 to 2014. Despite long-distance transport between hospitals in the acute phase of MI in our study, a different infrastructure and patient delay, long-term mortality rates were similar to those in other international studies.

### 4.1. Non-ST-Elevation Myocardial Infarction

The 5-year mortality after admittance for NSTEMI remained unchanged between 2014 and 2007. However, the time from admittance to PCI improved, whereas out-of-hospital time delays (symptoms to PCI) remained unchanged. Reducing prehospital time delay may be the best strategy to improve outcome, as increasing treatment delay was associated with higher risk of death in our study. It is therefore important to improve public awareness for early presentation. The treatment strategy in NSTEMI patients in our study followed updated ESC guidelines in the same period [16,17], which implies that an invasive strategy was favored [18] but highlights the role of risk stratification in the management decision process.

It is well documented that in patients with acute coronary syndrome, revascularization of the culprit artery soon after the onset of myocardial ischemia is associated with better immediate and long-term prognosis [19]. In both groups of NSTEMI in our study, approximately one third of patients had acute coronary occlusions, which was slightly higher compared to earlier studies which reported between proportions of 25–29% [20,21,22]. Total occlusion of infarct-related arteries increases mortality in patients with NSTEMI [21,23], and a strategy of very early invasive coronary evaluation does improve overall long-term clinical outcome compared with a delayed invasive strategy in patients with the highest risk [13,24]. Identifying these patients prior to angiography remains difficult. However, we found no difference in survival in NSTEMI patients with an occluded culprit artery. This could be due to high age, comorbidities and selection bias for invasive strategy in both groups.

Interestingly, despite a change in revascularization techniques favoring PCI over CABG, the rate of reinfarction was similar in both groups of NSTEMI patients. Furthermore, NSTEMI 2014 patients had lower risk of rehospitalization with heart failure compared to NSTEMI 2007. An explanation for this may be the lower rate of PCI among the NSTEMI 2007 group and improvement in heart failure treatment [25]. In addition, higher secondary medical treatment with ACE/A2 inhititors in NSTEMI 2014 patients contributed to the lower rate of rehospitalization with heart failure. In our study, the percentage of patients undergoing angiography/PCI or surgical revascularization was high compared to earlier studies [26,27].

LVEF has long been recognized as one of the most important predictors of mortality in patients with MI [28]. Lower LVEF was also associated with increased mortality in our study. GRACE risk score and Killip classification are simple and fast clinical tools for risk stratification of patients presenting with ACS. Heart failure presentation in our NSTEMI patients was recorded by Killip classification, which adds prognostic value to the study and may explain poor long-term prognosis [29]. High-risk NSTEMI patients are recommended to undergo an inpatient invasive strategy and should be considered for an early invasive strategy (i.e., within 24 h) [30]. Higher GRACE risk score in NSTEMI 2014 may have contributed to poor outcomes, despite the fact that a higher percentage of patients underwent an invassive strategy within 24 h and frequently received PCI. Furthermore, time delay from symptoms to invasive coronary angiography in NSTEMI patients remained unchanged after a period of ten years.

### 4.2. ST-Elevation Myocardial Infarction

Our study showed an improvement in survival after primary PCI compared with thrombolysis and rescue PCI in STEMI patients during the last decades. STEMI patients with primary PCI remained at lower risk of all-cause mortality and reinfarction after 5 years of follow-up. The gradual implementation of evolving treatments with reperfusion including primary PCI and medical therapy with antiplatelets may explain this prolonged survival. Time-delay from symptom onset to coronary angiography dramatically decreased, possibly constituting the most important change that improved survival. Not surprisingly, many STEMI 2004 patients initially treated with thrombolysis in 2004 did not achieve reperfusion and consequently the coronary artery remained occluded. Persistent coronary occlusion of the infarct-related artery impacts myocardial necrosis and influences on long-term survival [31].

NT-proBNP is closely linked to the extent of myocardial ischemia and cardiac function in MI patients and is associated with higher short- and long-term mortality in patients with acute coronary syndromes [32,33]. It is also well known that advanced age is a risk factor for impaired outcomes in patients with STEMI [34]. In our study, higher NT-proBNP, as well as advanced age, was also found to be a predictor of 5-year mortality after adjusting for other cardiovascular risk factors. Higher levels of troponin release in STEMI patients were an independent predictor of increased mortality after adjustment in multivariable analysis. According to the European guidelines, patients with a GRACE risk score of ≥140 are at high risk [17,30]. STEMI patients in our study with intermediate-high GRACE risk score showed strong predictive value for all-cause 5-year mortality.

The majority of STEMI patients had anterior wall infarction with left anterior descending artery (LAD) as the infarct-related artery. LAD as culprit lesion is associated with impaired LVEF and is strongly associated with adverse outcome [35]. Irreversible myocardial injury may be avoided by urgent reperfusion and might have implications in clinical outcomes. The higher rate of rehospitalization with heart failure in STEMI 2004 patients may in part be explained by delay in identification of these patients with occlusion after thrombolysis and needed further management. The impact of invasive strategies may differ according to the increased use of reperfusion therapy and recommended medications, and also to the organization of care aimed at shortening total ischemic time.

### 4.3. Clinical Implications

To improve outcomes in NSTEMI patients, it is crucial to rapidly identify those who have large infarctions with acute coronary occlusion. The findings in our study supports directing efforts to NSTEMI patients to improve the poor outcomes seen regardless of the chosen management strategy. Patients with potential NSTEMI should be hospitalized immediately and assessed by cardiac biomarkers and bedside echocardiography [13]. Patients with findings suggestive of occluded infarct-related arteries should be assessed by urgent coronary angiography without delay when dynamic unstable chest pain or ECG changes are found.

Patients with NSTEMI appear to receive similar medical treatment after discharge from hospital regardless of their risk profile. Interestingly, reinfarction rate was unchanged in the NSTEMI groups but decreased in the STEMI groups. This finding might suggest that NSTEMI patients should be more aggressively treated with secondary prevention to avoid reinfarctions. Despite differences in presentation and different management strategies in patients with NSTEMI versus STEMI, both types must be considered as equally dangerous. Our study adds to the evidence that effective and rapid revascularization is important to achieve improved clinical outcomes in STEMI patients. Furthermore, a time-delay may be associated with increased mortality not only in STEMI patients but also in NSTEMI patients.

### 4.4. Study Limitations

Our study had several limitations that should be acknowledged. The current study was part of an echocardiographic observational study with inherent limitations, such as selection bias and confounding. We did not have longitudinal follow-up with changes in medical therapy after initial hospital discharge. Echocardiography was performed in an early stage of AMI with developing edema that may be accompanied by alterations in LVEF. We did not record antiplatelet treatment duration or compliance, which may have influenced reinfarction rates. However, it is unlikely to be profoundly different in the two time periods.

The data for this patient population were from a prospective echocardiographic study and lacked precise calculation for an epidemiologic study; thus, the sample size was relatively small for epidemiologic purposes and inadequate to attempt propensity score matching. There is lack of gender specific adjustments in prediction of primary endpoints that may be important in patients with ACS [36].

To perform intravascular ultrasound (IVUS) and fractional flow reserve (FFR) procedures was not part of this study protocol. However, earlier studies showed that IVUS and FFR during coronary angioplasty in patients with ACS was not associated with better short- or long-term survival [37].

Some angiography-specific findings, such as TIMI flow pre- and post-PCI, were not recorded, which may potentially explain some of the factors that influenced mortality. Intra-aortic balloon pump placement occurred in the study population with cardiogenic shock but was not part of our study protocol. As the definition of cardiogenic shock has not remained consistent in the past twenty years, this data could not be included. However, the use of intraaortic balloons, as compared with conventional therapy, did not reduce mortality [38].

All patients were managed in a single center, and the results of our study may not be valid for other regions or in regions with other organizations of AMI care. Another potential limitation is the higher rate of surgical revascularization in patients assigned to earlier groups, which may have affected the outcomes due to CABG and potential complications of surgical revascularization.

## 5. Conclusions

The main finding of our study was that after one decade of AMI treatment, mortality in patients with NSTEMI remained unchanged while mortality in STEMI patients decreased, both at 1 and 5 years. Possible reasons for improvement of outcomes in STEMI patients are due to reduction in ischemic time and optimal medical therapy. Occurrence of reinfarction and heart failure rehospitalizations in STEMI patients decreased, while the rate of reinfarction in NSTEMI patients remained unchanged. Further appropriate selection of NSTEMI patients at risk for immediate revascularization may potentially improve long-term mortality.

## Figures and Tables

**Table 1:** Baseline characteristics in 539 NSTEMI and STEMI patients.

	NSTEMI 2014	NSTEMI 2007	*p*-Value	STEMI 2014	STEMI 2004	*p*-Value
	(*n* = 234)	(*n* = 82)		(*n* = 160)	(*n* = 63)	
Baseline clinical characteristics						
Age, years	67 ± 13	63 ± 14	0.06	63 ± 11	66 ± 12	0.10
Age > 65, no (%)	136 (58)	37 (45)	0.04	67 (42)	32 (51)	0.20
Male/female, n:n	173:61	61:21	0.50	125:35	49:14	0.50
BP, diastolic, mmHg	77 ± 12	71 ± 11	<0.001	76 ± 11	74 ± 19	0.50
BP, systolic, mmHg	131 ± 19	127 ± 15	0.90	123 ± 17	139 ± 25	<0.001
Hypertension, no (%)	114 (49)	36 (44)	0.50	65 (41)	18 (29)	0.10
Diabetes mellitus, no (%)	36 (16)	7 (9)	0.10	28 (18)	4(6)	0.03
CAD history, no (%)	61 (26)	10 (12)	0.009	23 (14)	6 (10)	0.40
Troponin T, ng/L (IQR)	508 (180–1523)	830 (240–2385)	0.10	3399 (1343–7165)	4990 (1535–10,257)	0.10
NT-pro BNP, ng/L (IQR)	209 (30–239)			337 (62–399)		
Heart rate, bpm	70 ± 13	66 ± 10	0.004	73 ± 11	74 ± 19	0.50
Killip classification, II-IV, no(%)	30 (13)	6 (7)	0.10	29 (18)	14 (22)	0.30
GRACE risk score	109 ± 28	104 ± 28	0.04	118 ± 25	121 ± 27	0.40
Medication						
Aspirin, no (%)	224 (97)	82 (100)	0.40	158 (99)	58 (95)	0.07
Clopidogrel/Ticagrelor, no (%)	228 (97)	82 (100)	0.40	159 (100)	54 (89)	<0.001
B-blockers, no (%)	173 (76)	71 (87)	0.04	143 (90)	58 (95)	0.30
Statins, no (%)	213 (93)	81 (99)	0.05	145 (98)	58 (94)	0.20
ACE or ARBs, no (%)	95 (43)	16 (20)	<0.001	100 (64)	41 (67)	0.80
Echocardiography						
LVEF, %	49 ± 8	49 ± 6	0.90	46 ± 7	45 ± 10	0.50
LVESV, mL	70 ± 30	69 ± 22	0.90	68 ± 24	58 ± 25	0.009
LVEDV, mL	135 ± 39	134 ± 31	0.90	125 ± 36	106 ± 32	<0.001
Time to revascularization						
Symptoms to thrombolysis, hours (IQR)					2.5 (1.4–3.9)	
Hospitalization to PCI/CABG, hours (IQR)	14.2 (8.2–22.4)	21.8 (15.8–37.5)	<0.001	0.2 (0.2–0.47)	25.9 (3.3–29.2)	<0.001
Symptoms to coronary angiography, hours (IQR)	28.2 (18.1–46.3)	30.3 (18.0–48.3)	0.20	2.8 (2.0–4.8)	21.7 (5.4–27.1)	<0.001
Early revascularization, <24 h (%)	175 (79)	49 (60)	<0.001	159 (100)	35 (70)	<0.001

Values are mean ± SD or numbers (percentage) or median with interquartile range (IQR); *p*-values are calculated from unpaired Student’s *t*-test; Mann–Whitney U-test and Chi-square when appropriate. ACE = angiotensin-converting enzyme; ARBs = angiotensin II receptor blockers; BP = blood pressure; Bpm = beats per minute; CABG = coronary artery bypass graft; CAD = coronary artery disease; ECG = electrocardiogram; GRACE = Global Registry of Acute Coronary Event; LV = left ventricular, LVEF = left ventricular ejection fraction; LVESV = left ventricular end-systolic volume; LVEDV = left ventricular end-diastolic volume; No = number; NSTEMI = non-ST-elevation myocardial infarction; NT-pro BNP = N-terminal pro-B-type natriuretic peptide; PCI = percutaneous coronary intervention; STEMI = ST-elevation myocardial infarction.

**Table 2:** Primary endpoint and revascularization results in the NSTEMI populations.

	NSTEMI 2014 *(n* = 234)	NSTEMI 2007 (*n* = 82)	*p* Value
Primary outcome			
1-year all-cause mortality, no (%)	8 (3.5)	4 (4.9)	0.50
5-year all-cause mortality, no (%)	26 (11.4)	12 (14.6)	0.40
Secondary outcome			
AMI recurrence, no (%)	26 (11)	15 (18)	0.10
Rehospitalization with heart failure, no (%)	7 (4)	9 (11)	0.02
Culprit lesion			
Stenosis, no (%)	156 (67)	51 (62)	0.50
Occluded, no (%)	78 (33)	31 (38)	0.50
Left anterior descending artery, no (%)	99 (42)	26 (32)	0.06
Circumflex artery, no (%)	65 (28)	22 (27)	0.50
Right coronary artery, no (%)	57 (24)	24 (29)	0.40
Revascularization			
Percutaneous coronary intervention, no (%)	218 (93)	69 (84)	0.02
Coronary artery bypass graft, no (%)	3 (1)	7 (9)	0.004
No intervention, no (%)	13 (6)	6 (7)	0.60

Values are mean ± standard deviation. AMI = acute myocardial infarction; NSTEMI = non-ST-elevation myocardial infarction; PCI = percutaneous coronary intervention.

**Table 3:** Primary endpoint and revascularization results in the STEMI populations.

	STEMI 2014(*n* = 160)	STEMI 2004 (*n* = 63)	*p* Value
Primary outcome			
1-year all-cause mortality, no (%)	2 (1.3)	7 (11.1)	<0.001
5-year all-cause mortality, no (%)	11 (7.0)	14 (22.2)	0.004
Secondary outcome			
AMI recurrence, no (%)	6 (4)	16 (25)	<0.001
Rehospitalization with heart failure, no (%)	1 (1)	9 (15)	<0.001
Culprit lesion			
Stenosis, no (%)	36 (23)	35 (56)	<0.001
Occluded, no (%)	124 (76)	25 (40)	<0.001
Left anterior descending artery, no (%)	75 (47)	34 (54)	0.20
Circumflex artery, no (%)	19 (12)	7 (11)	0.60
Right coronary artery, no (%)	62 (39)	19 (28)	0.10
Revascularization			
Rescue PCI, no (%)		17 (27)	
PCI, no (%)	157 (98)	52 (84)	<0.001
Coronary artery bypass graft, no (%)	0 (0)	5 (8)	0.002
No intervention, no (%)	3 (2)	5 (8)	0.04

Values are mean ± standard deviation. No = number. AMI = acute myocardial infarction; PCI = percutaneous coronary intervention; STEMI = ST-elevation myocardial infarction.

**Table 4:** Uni- and multivariable analyses of independent predictors for 5-year mortality in NSTEMI patients and STEMI patients.

	NSTEMI				STEMI			
	Univariable HR (95% CI)	*p*-Value	Multivariable HR (95% CI)	*p*-Value	Univariable HR (95% CI)	*p*-Value	Multivariable HR (95% CI)	*p*-Value
Risk factor								
Age (per year increment)	1.11 (1.07–1.15)	<0.001	1.09 (1.05–1.13)	<0.001 *	1.10 (1.06–1.15)	<0.001	1.12(1.06–1.19)	<0.001 *
BMI	0.90 (0.80–0.97)	0.01	0.94 (0.87–1.04)	0.20 *	0.91 (0.80–1.05)	0.20		
Diabetes mellitus	1.20 (0.50–2.90)	0.70			1.07 (0.32–3.63)	0.90		
Smoker/prior smoker	0.66 (0.44–0.98)	0.04	0.91 (0.60–1.43)	0.70 *				
CAD history	3.30 (1.70–6.30)	<0.001	2.08 (1.05–4.10)	0.03 *	2.49 (0.88–6.30)	0.05	0.7 (0.13–3.80)	0.70 *
Clinical features								
GRACE risk score > 140	5.65 (2.98–10.73)	<0.001	6.26 (2.19–17.91)	<0.001	6.27 (2.85–13.81)	<0.001	6.61 (2.5–17.47)	<0.001
NT-proBNP (per 10-fold increase)	2.86 (1.49–5.48)	0.002	0.74 (0.29–1.86)	0.50	1.9.30 (4.96–75.12)	<0.001	24.70 (2.75–221.2)	0.004
Troponin T (per 10-fold increase)	1.01 (0.59–1.73)	0.90			5.40 (1.79–16.30)	0.003	4.9 (1.10–21.93)	0.04
LVEF	0.93 (0.90–0.96)	<0.001	4.56 (1.54–13.50)	<0.006	0.94 (0.90–0.98)	0.008	0.97 (0.92–1.03)	0.40
Heart rate	1.03 (1.00–1.06)	0.04	1.05 (1.05–1.09)	0.003	1.02 (0.99–1.05)	0.08		
Hospitalization to PCI, hour (per 10-fold increase) ◊	5.60 (1.99–15.74)	<0.001	4.03 (1.26–12.89)	0.02	2.34 (1.05–5.24)	0.04	2.12 (1.05–4.28)	0.04
Symptoms to PCI, hour (per 10-fold increase) ◊	3.43 (1.32–8.90)	0.01	0.22 (0.655–6.26)	0.05 ¥	2.95 (1.30–6.73)	0.01	2.48 (1.10–5.58)	0.03 ¥

Hazard ratio (HR) by uni- and multivariable regression. HR is calculated for 1 year increase of age, per 1% decrease in LVEF. ◊ Log base-10 transformation to meet model linearity assumptions. CAD = coronary artery disease; BMI = body mass index; CI = confidence interval; GRACE = Global Registry of Acute Coronary Event; LVEF = left ventricle ejection fraction; MI = myocardial infarction; NSTEMI = non-ST-elevation myocardial infarction; PCI = percutaneous coronary intervention; STEMI = ST-elevation myocardial infarction. * Multivariable analyses including age, BMI, diabetes mellitus and CAD history. Multivariable analysis including NT-proBNP, troponin T, LVEF, heart rate and hospitalization to PCI. ¥ Multivariable analysis including troponin T, LVEF, heart rate and symptoms to PCI.

## Data Availability

The data presented in this study were obtained from the Department of Cardiology, Hospital of Southern of Norway and are not openly available.
